# The Practice of Therapeutic Hypothermia after Cardiac Arrest in France: A National Survey

**DOI:** 10.1371/journal.pone.0045284

**Published:** 2012-09-25

**Authors:** Jean-Christophe Orban, Florian Cattet, Jean-Yves Lefrant, Marc Leone, Samir Jaber, Jean-Michel Constantin, Bernard Allaouchiche, Carole Ichai, for the AzuRéa group

**Affiliations:** 1 Réanimation médico-chirurgicale, Hôpital Saint-Roch, Centre Hospitalier Universitaire de Nice, Nice, France; 2 IRCAN, Faculté de Médecine, Université de Nice, Nice, France; 3 Division Anesthésie Réanimation Douleur Urgences, Centre Hospitalier Universitaire de Nîmes, Nîmes, France; 4 Service anesthésie et réanimation, Hôpital Nord, Marseille, France; 5 Département d’Anesthésie et de Réanimation, Centre Hospitalier Universitaire de Montpellier, Montpellier, France; 6 Unité de Soins Intensifs, Centre Hospitalier Universitaire de Clermont-Ferrand, Clermont-Ferrand, France; 7 Département d’Anesthésie-Réanimation, Hôpital Edouard-Herriot, Hospices Civils de Lyon, Lyon, France; S.G.Battista Hospital, Italy

## Abstract

**Aims:**

Cardiac arrest is a major health concern worldwide accounting for 375,000 cases per year in Europe with a survival rate of <10%. Therapeutic hypothermia has been shown to improve patients’ neurological outcome and is recommended by scientific societies. Despite these guidelines, different surveys report a heterogeneous application of this treatment. The aim of the present study was to evaluate the clinical practice of therapeutic hypothermia in cardiac arrest patients.

**Methods:**

This self-declarative web based survey was proposed to all registered French adult intensive care units (ICUs) (n = 357). Paediatrics and neurosurgery ICUs were excluded. The different questions addressed the structure, the practical modalities of therapeutic hypothermia and the use of prognostic factors in patients admitted after cardiac arrest.

**Results:**

One hundred and thirty-two out of 357 ICUs (37%) answered the questionnaire. Adherence to recommendations regarding the targeted temperature and hypothermia duration were 98% and 94% respectively. Both guidelines were followed in 92% ICUs. During therapeutic hypothermia, sedative drugs were given in 99% ICUs, mostly midazolam (77%) and sufentanil (59%). Neuromuscular blocking agents (NMBA) were used in 97% ICUs, mainly cisatracurium (77%). Numerous prognostic factors were used after cardiac arrest such as clinical factors (95%), biomarkers (53%), electroencephalography (78%) and evoked potentials (35%).

**Conclusions:**

In France, adherence to recommendations for therapeutic hypothermia after cardiac arrest is higher than those previously reported in other countries. Numerous prognostic factors are widely used even if their reliability remains controversial.

## Introduction

Cardiac arrest is a major health concern in industrialised countries accounting for 375000 cases per year in Europe with an overall survival <10% [1], [2], [3]. Resuscitation is considered successful when patients exhibit a good neurological outcome (usually cerebral performance category 1 and 2 corresponding to good cerebral performance or moderate disability). Among numerous interventions assessed to improve neurological outcome, only therapeutic hypothermia obtained good results after cardiac arrest due to ventricular fibrillation [4], [5]. In the study by Bernard et al., mild hypothermia at 33°C during 12 hours improved survival with a good outcome (adjusted odds ratio for good outcome: 5.25 [95% confidence interval 1.47 to 18.76]) [4]. In the HACA study, therapeutic hypothermia with a target temperature between 32 and 34°C during 24 hours showed comparable findings with a decreased mortality (risk ratio: 0.74 [95% confidence interval 0.58 to 0.95]) and a favourable neurologic outcome (risk ratio: 1.40 [95% confidence interval 1.08 to 1.81]) [5]. Further retrospective studies reported similar benefit when therapeutic hypothermia was applied in patients with cardiac arrest secondary to initial ventricular fibrillation [6], [7], [8]. Such findings were not reported in patients with initial asystole [8].

Based on these prospective studies, the European Resuscitation Council (ERC) published recommendations in 2005, updated in 2010, for the use of mild therapeutic hypothermia in unconscious adult patients with spontaneous circulation after out-of-hospital cardiac arrest when the initial rhythm is ventricular fibrillation but also asystole [9]. The statement recommends duration of therapeutic hypothermia of 12 to 24 hours and a temperature between 32 and 34°C. Despite the high evidence of these recommendations, different surveys reported the application of therapeutic hypothermia in 13% to 61% [10]–[14]. As the application of therapeutic hypothermia after cardiac arrest has not been assessed in France, a national survey was aimed at evaluating the management of this treatment in French intensive care units (ICUs).

## Materials and Methods

Our institutional review board (Comité de protection des personnes Sud Méditerranée V) has waived the need for ethical approval for this study. Addresses of all registered French intensive care units (*n* = 357) were obtained from the national health facilities website. Paediatrics and neurosurgery ICUs were excluded of the study. A structured evaluation questionnaire was sent to the heads of the ICU (medical or surgical intensivist) by e-mail starting October 2010 to May 2011 (see Questionnaire S1). The survey addressed structure of the hospitals, number of admissions for cardiac arrest in 2009, indications for the use of therapeutic hypothermia after cardiac arrest, cooling methods employed, sedation practices and prognostic factors used (see additional material). When no response was received after 2 reminders, ICUs clinical managers were contacted by phone to increase the rate of responses. Data collection ended in June 2011.

Quantitative and qualitative data are expressed in median value with interquartile range (IQR) and in frequency with percentage (%) with the 95% confidence interval.

## Results

One hundred and thirty two questionnaires were returned (response rate = 37% [32–42%]), mainly from non-academic institutions (n = 81, 61%). Six units declared not using therapeutic hypothermia, leaving 126 units for final analysis of these declarative data.

Indication of hypothermia:

Therapeutic hypothermia was induced after out of hospital cardiac arrest due to ventricular fibrillation in 119 ICUs (99% [97–101%]) and after asystole in 111 ICUs (93% [88–98%]).

### Method of Cooling

External, internal and mixed methods were applied in 54% [45–63%], 20% [13–27%] and 26% [19–35%] ICUs, respectively. The main reasons for this choice were the easiness of applicability (64% [55–73%]) and the cost effectiveness (48% [39–57%]). The time to reach the target temperature was assessed as <6 hours in 92 ICUs (75% [67–83%]). Temperature was monitored by oesophageal (49% [40–58%]) or bladder catheter probes (40% [31–49%]), thermometer (11% [5–17%]), skin and rectal probes (21% [14–28%]).

**Figure 1:**
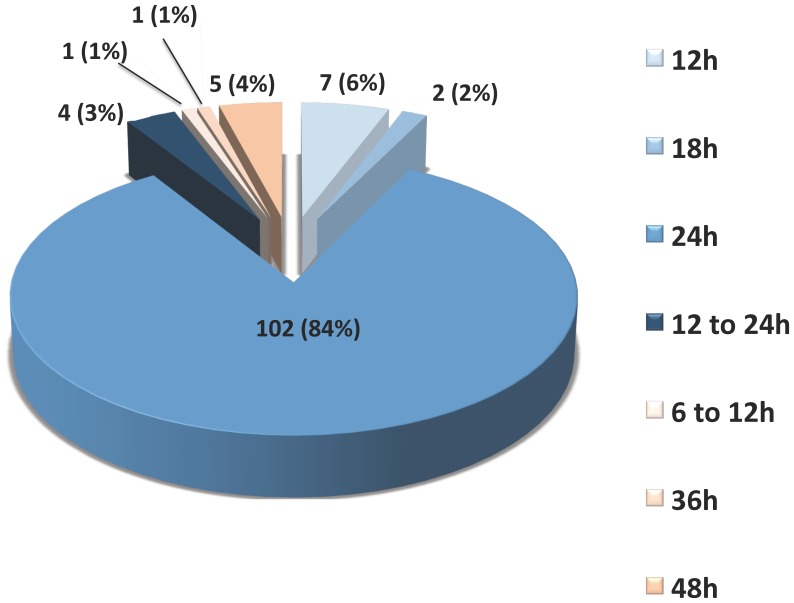
Practice of hypothermia duration. Figure sectors in light blue to dark blue are in the recommended target. Figure sectors in light orange to orange are outside the recommended target.

**Figure 2:**
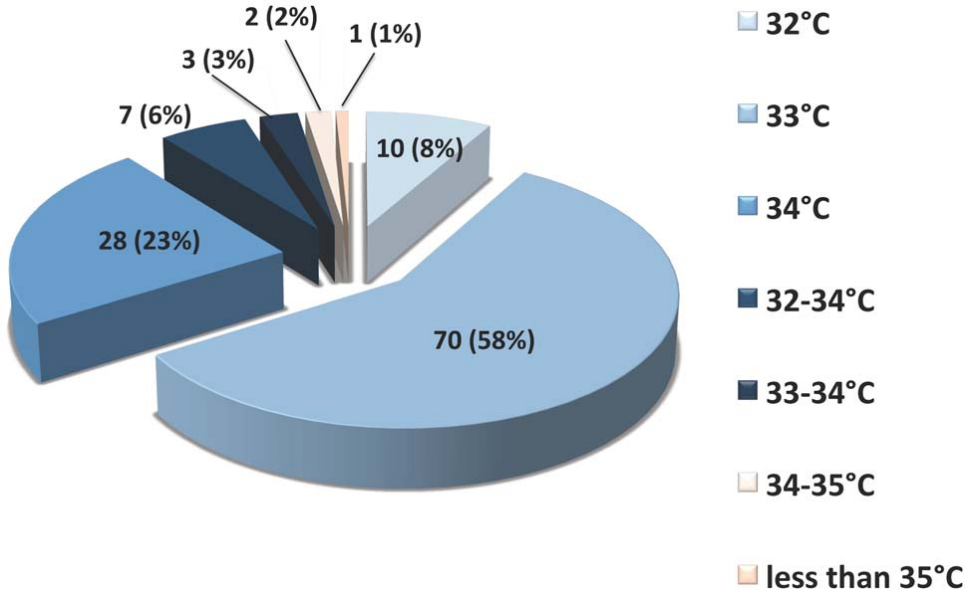
Practice of hypothermia target temperature. Figure sectors in light blue to dark blue are in the recommended target. Figure sectors in light orange to orange are outside the recommended target.

Target temperature and duration of hypothermia (figures 1 and 2):

The targeted temperatures were 33°C (58% [49–67%]), 34°C (23% [16–30%]) and 32°C (8% [3–13%]). Less frequently, the units chose a range of temperature between 32 and 34°C (6% [2–10%]), and between 33 and 34°C (3% [0–6%]). Overall, the ERC recommendations regarding the targeted temperature (32 to 34°C) were followed in 118 ICUs (98% [96–100%]). Few units were outside the recommended temperature range: between 34 and 35°C (n = 2, 2% [0–4%]) and less than 35°C (n = 1, 1% [−1–3%]). The planned duration of therapeutic hypothermia was 24 hours in 101 ICUs (84% [77–91%]), 12 h in 7 ICUs (6% [2–10%]), 12 to 24 h in 4 ICUs (3% [0–6%]) and 18 h in 2 ICUs (2% [0–4%]), leading to an implementation of the ERC recommendations in 114 units (94% [90–98%]). The remaining expressed durations of hypothermia were 48 h (n = 5, 4% [0–8%]), 36 hours (n = 1, 1% [−1–3%]) and 6 to 12 h (n = 1, 1% [−1–3%]). Concerning both the targeted temperature and the duration of hypothermia, 112 ICUs (92% [87–97%]) followed both ERC recommendations.

### Sedation during Therapeutic Hypothermia

During therapeutic hypothermia, patients were given sedative drugs in 99% [97–101%] of ICUs. Midazolam (77% [70–84%]) and propofol (10% [5–15%]) were the hypnotic drugs used in first line. Sufentanil (59% [50–68%]), fentanyl (25% [17–33%]) or remifentanil (11% [6–16%]) were the most used analgesic drugs. Neuromuscular blocking agents were given during therapeutic hypothermia in 97% [94–100%] of units, mainly cisatracurium (77% [70–84%]) and atracurium (16% [10–22%]).

### Prognostic Factors

One hundred and twenty ICUs (95% [91–99%]) reported the utilisation of one or more clinical prognostic factors such as absence of brainstem reflexes (90% [85–95%]), duration of cardiopulmonary resuscitation (85% [79–91%]), fixed dilated pupils (75% [67–83%]), seizures (73% [65–81%]) and the Glasgow Coma Scale (3% [0–6%]). Sixty-seven units (53% [44–62%]) used biological prognostic factors, mainly lactate (55% [43–67%]) and Neuron Specific Enolase (31% [20–42%]). The use of a combination of several biomarkers was reported in 13% [5–21%] of ICUs. Evoked potentials were used in 44 ICUs, somesthetic in 77% [65–89%] and auditory in 23% [11–35%]. Ninety-eight ICUs reported the use of electroencephalography; flat trace (87% [80–94%]) and burst suppression pattern (58% [48–68%]) were the most frequent prognostic factors. Withdrawal of life sustaining treatments was considered in 98% [96–100%] of ICUs.

## Discussion

In this declarative survey involving 126/353 of French ICUs, therapeutic hypothermia was induced after out of hospital cardiac arrest due to ventricular fibrillation and after asystole in 99% and 93% of ICUs respectively. The European Resuscitation Council recommendations regarding the target and the duration of therapeutic hypothermia were followed in 98% and 94% ICUs. Adherence to these two major objectives was 92% ICUs. Sedative and neuromuscular blocking drugs were given in 99% and 97% ICUs, respectively. Clinical, biological and electroencephalographic prognostic factors were searched in 95%, 53% and 78% ICUs respectively.

In our study, the percentage of compliance to recommendations regarding target temperature and hypothermia duration is higher compared to the figures reported previously in the United-Kingdom (63% for temperature and 72% for duration) [12]. Compliance to the 2 major recommendations is similar to the 83% previously reported in Italy [14]. Moreover, the rate of implementation of ERC guidelines is higher than those reported for the application of other recommendations such as protective ventilation during acute lung injury (50%) and initial management of severe sepsis and/or septic shock [15], [16]. This difference could be explained by an easier identification of cardiac arrest patients. Furthermore, the ERC recommendations are not numerous and could be easily performed. Indeed, as several barriers could explain the poor implementation of guidelines, providing precise and simple recommendation is likely to facilitate their implementation by physicians [17].

The range of recommended temperature is broad and the most efficacious target is still unknown. A more precise targeted temperature could be associated with different efficacy and complications. Only one study addressed this question comparing the effects of 32°C, 33°C and 34°C as target temperature after cardiac arrest [18]. Unfortunately, this work showed no difference between the different groups, probably due to a lack of power. Interestingly, only few ICUs use a range of temperature whereas most of them prefer to set a precise target in this range.

External cooling is the most common method to induce therapeutic hypothermia. This is in agreement with previous reports [10], [11], [14]. However, internal cooling is increasingly performed. Compared to conventional external cooling, intravascular methods provides a target temperature faster, a more stable hypothermia and a better control of rewarming [19], [20]. This difference is no longer present between modern surface cooling devices and core devices [21]. The type of cooling method has no influence on mortality as confirmed by the large prospective study ICEREA [22].

Our work reports the sedation practice during therapeutic hypothermia after cardiac arrest in France. Midazolam is the most common sedative drug in agreement with a previous report of worldwide practice [23]. In the United-Kingdom, propofol and midazolam are given in the same proportion [12]. To date, there are no recommendations for the first choice sedative drug in this setting. Compared to midazolam, pharmacokinetics of propofol is not altered by hypothermia. Moreover, its antioxidant properties could be of clinical interest to mitigate the post-resuscitation syndrome [24]. Sufentanil is the most commonly used opioid during therapeutic hypothermia after cardiac arrest in France, whereas worldwide, fentanyl is the first choice. However, fentanyl has been shown to accumulate during hypothermia [25]. There is no data available concerning the pharmacokinetics of sufentanil during hypothermia. As it is metabolized by the same cytochrome, hypothermia may also increase its half-life. It is advisable to give NMBA during therapeutic hypothermia and this recommendation is followed by 97% of French ICUs. This percentage is higher than the 79% reported in the rest of the world by Chamorro et al [23]. The most commonly used NMBA in France is cisatracurium whereas pancuronium is the first choice worldwide. This difference can be explained by the withdrawal of pancuronium from the French market and cisatracurium is supposed to be the only NMBA authorized in ICUs in France. Similarly to the sedative drugs, the question of the best NMBA is still unanswered. However, the recent report of a better outcome with the use of cisatracurium in patients with ARDS could partly explain the easy use of cisatracurium in ICU setting [26].

Prognostic factors are frequently used, mostly neurological examination and EEG studies and to a lesser extent biomarkers and evoked potentials. Guidelines concerning the use of prognostic factors were made before the era of therapeutic hypothermia [27]. Since this treatment is recommended after out of hospital cardiac arrest, the validity of these factors is questioned [28]. Indeed, hypothermia prolongs sedation as it impairs the metabolism of the most common sedative and analgesic drugs such as midazolam and fentanyl. A recent study failed to find a clinical difference between hypothermic and non-hypothermic patients regarding the time of awakening, but the infusion regimen of sedative drugs was very low compared to previous reports [23], [29]. Currently, the use of prognostic factors raises crucial questions. The first concern is the cost of these additional tests as resources are becoming scarce in most ICUs. The second concern is ethical with possible premature withdrawal of life sustaining therapies. Further recommendations are urgently needed to clarify the use of the prognostic factors after cardiac arrest treated by hypothermia.

While offering novel insight, our study has to be interpreted with caution. The first caveat is the percentage of ICUs answering of only 37%, questioning the validity of our results. Rates of answer of previous surveys on therapeutic hypothermia after cardiac arrest range from 17 to 98.4% [11], [12]. The type of survey could explain this apparent difference as a survey delivered by post has a higher percentage of answer than a survey delivered by e-mail [30]. Actually, the percentage of answers of our study is higher compared to the study using the same type of survey [10], [11]. Another limit of our study lies in the type of a self-declaration survey. There is probably a discrepancy between what is declared and what is done in “real-life”. An audit realized by a trained staff in the different units could diminish this potential bias. Last, our study was focused on therapeutic hypothermia. But recently, percutaneous coronary intervention (PCI) has been reported to improve outcome after out of hospital cardiac arrest [31]. Data concerning its practice was not included in the questionnaire so it was impossible to report the combination of both therapies. Further studies are needed to evaluate the practice of a postresuscitation care bundle and its effect on outcome after out of hospital cardiac arrest.

### Conclusions

Despite a low response rate of the survey, practice of therapeutic hypothermia after cardiac arrest follows the international guidelines in France. Prognostic factors are widely used but their value in the era of therapeutic hypothermia is still debated.

## Supporting Information

Questionnaire S1 File sent by e-mail to the head of the ICUs for the purpose of the survey.
(DOC)
