# Supplementary material for: The Practice of Therapeutic Hypothermia after Cardiac Arrest in France: A National Survey
Source: PLoS One. 2012 Sep 25;7(9):e45284. doi: 10.1371/journal.pone.0045284 (PMC3458038; doi:10.1371/journal.pone.0045284)
Supplement: Questionnaire S1 — File sent by e-mail to the head of the ICUs for the purpose of the survey. (DOC) [file pone.0045284.s001.doc]

**Questionnaire on therapeutic hypothermia practice and outcome after cardiac arrest**

- **What is your type of hospital?**

**General hospital**  **University hospital**  **Private hospital**  **Private hospital participating to public service**

- **How many ICU beds have you in your unit? _ _ _**
- **How many admissions had you in 2009 ? _ _ _**
- **How many admissions for out of hospital cardiac arrest had you in 2009 ? _ _ _**
- **What is the type of your ICU?**

**Surgical**  **Medical**  **Surgical and medical**  **Other**

- **Do you induce therapeutic hypothermia after cardiac arrest?**

**If no, can you specify why: _ _ _ _ _**

**If yes, specify in which case:**

- **Out of Hospital Cardiac Arrest**
  - **Ventricular fibrillation**  **YES**  **NO**
  - **Asystole**  **YES**  **NO**
- **In Hospital Cardiac Arrest**
  - **Ventricular fibrillation**  **YES**  **NO**
  - **Asystole**  **YES**  **NO**
- **Which cooling method do you use?**

**External (specify) :**

**Internal (specify) :**

- **What is the reason of this choice?**

**Economical**  S**cientific**  **Practical**  **other (specify) :**

- **What is your mean duration to reach target temperature ?**

**0 to 6h**  **6 to 12h**  **12 to 24h**  **> 24h**

- **What is your target temperature?**

**32°c**  **33°c**  **34°c**  **Other (specify) :**

- **What is your duration of therapeutic hypothermia?**

**12h**  **24h**  **Other (specify) :**

- **How do you monitor temperature during hypothermia?**

**Bladder catheter**  **Esophageal probe**  **Thermometer**

**Other (specify) :**

- **Do you sedate the patient during hypothermia?**

**YES**  **NO**

**If YES, which sedative drug do you use?**

**Midazolam**  **Propofol**

**Other (specify) :**  **No sedative drug**

**If YES, which opioid do you use?**

**Morphine**  **Fentanyl**  **Sufentanil**

**Remifentanil**  **Other (specify) :**  **No opioid**

**If YES, which neuromuscular blocking agent do you use?**

**Atracurium**  **Cis-atracurium**

**Other (specify) :**  **No NMBA**

- **Which rewarming method do you use?**

**Passive (specify) :**

**Active (specify) :**

- **Which prognostic factors do you use?**

**Clinical (specify) :**

**Cardiopulmonary resuscitation duration (no flow, low flow)**

**Absence of brainstem reflexes**

**Absence pupillary reactivity**

**Seizures**

**Biomarkers (specify) :**

**Lactate**

**Neuron Specific Enolase**

**Other: _ _ _**

**Evoked potentials (specify) :**

**Somesthetic**

**Auditory**

**EEG (specify) :**

**Flat** **EEG**

**Spikes and waves**

**Burst suppression**

**Alpha oscillations**

**No prognostic factors**

- **Concerning patient outcome do you consider a possibility of life sustaining treatments withdrawal? YES/NO**
